# Fibro-Vascular Coupling in the Control of Cochlear Blood Flow

**DOI:** 10.1371/journal.pone.0020652

**Published:** 2011-06-01

**Authors:** Min Dai, Xiaorui Shi

**Affiliations:** 1 Oregon Hearing Research Center, Department of Otolaryngology/Head and Neck Surgery, Oregon Health & Science University, Portland, Oregon, United States of America; 2 The Institute of Microcirculation, Chinese Academy of Medical Sciences and Peking Union Medical College, Beijing, China; 3 Department of Otolaryngology, Renji Hospital, Shanghai Jiao Tong University, Shanghai, China; University of Arizona, United States of America

## Abstract

**Background:**

Transduction of sound in the cochlea is metabolically demanding. The lateral wall and hair cells are critically vulnerable to hypoxia, especially at high sound levels, and tight control over cochlear blood flow (CBF) is a physiological necessity. Yet despite the importance of CBF for hearing, consensus on what mechanisms are involved has not been obtained.

**Methodology/Principal Findings:**

We report on a local control mechanism for regulating inner ear blood flow involving fibrocyte signaling. Fibrocytes in the super-strial region are spatially distributed near pre-capillaries of the spiral ligament of the albino guinea pig cochlear lateral wall, as demonstrably shown in transmission electron microscope and confocal images. Immunohistochemical techniques reveal the inter-connected fibrocytes to be positive for Na+/K+ ATPase β1 and S100. The connected fibrocytes display more Ca^2+^ signaling than other cells in the cochlear lateral wall as indicated by fluorescence of a Ca^2+^ sensor, fluo-4. Elevation of Ca^2+^ in fibrocytes, induced by photolytic uncaging of the divalent ion chelator *o*-nitrophenyl EGTA, results in propagation of a Ca^2+^ signal to neighboring vascular cells and vasodilation in capillaries. Of more physiological significance, fibrocyte to vascular cell coupled signaling was found to mediate the sound stimulated increase in cochlear blood flow (CBF). Cyclooxygenase-1 (COX-1) was required for capillary dilation.

**Conclusions/Significance:**

The findings provide the first evidence that signaling between fibrocytes and vascular cells modulates CBF and is a key mechanism for meeting the cellular metabolic demand of increased sound activity.

## Introduction

Sound stimulation applied to the inner ear imposes an energy demand that requires delivery of oxygen and glucose, a demand that requires a well-regulated cochlear blood flow (CBF) to provide both substrate and efficacious clearance of metabolic products. While decades of studies from different laboratories have shown that moderate sound activity significantly increases red blood cell velocity, dilates vessels, and decreases local oxygen pressure [1], [2], [3], [4], the underlying physiological mechanisms remain undefined.

Regulation of CBF, under the prevailing model, is hypothesized to include both local auto-regulation and central control via neuronal pathways. In particular, CBF is thought to be mainly regulated in the end arterial system of the cochlea, specifically in the spiral modiolar artery and its branching arterioles [5], [6]. The model incorporates neural- and autocrine/paracrine-based regulation of vasoconstriction and dilation at the level of the artery and arterioles [5], [7]. Capillary-mediated local control of perfusion has been less studied. Our recent findings showing that cochlear capillaries are densely populated by pericytes expressing contractile proteins [8] and exhibiting vasocontractility [9] reopens the question about the role of capillary-based local blood-flow control.

The cochlea has two microvessel networks: the capillaries of the stria vascularis and spiral ligament [10]. Both capillary networks are located in the cochlear lateral wall, anatomically distant (>100 micrometers) from sensory hair cells in the organ of Corti, an arrangement that minimizes the effect of perturbations in blood flow on hearing. The capillaries of the spiral ligament are arterio-venular anastomosing vessels, passing directly across the ligament. Preliminary studies suggest that regulation of the CBF is dominated by this system [11]. In contrast, the capillaries of the stria vascularis form a blood-labyrinth barrier that is critical for maintaining endocochlear potential (EP), ion transport, and fluid balance in the inner ear [12], [13], [14]. The EP is necessary for sensory hair cell transduction.

Regulatory vessels of the spiral ligament are surrounded by five types of fibrocytes (I–V), categorized on the basis of morphological appearance, immunostaining pattern, and general location (see Fig. S1) [15], [16], [17]. Since fibrocytes participate in ion transport and facilitate generation of the EP by recycling K^+^ from hair cells to intermediate cells in the stria vascularis through gap junctions [13], [18], [19], increased hair-cell activity must be matched by increased fibrocyte metabolism. In brain and retina, similar cells (astrocytes and glial cells) couple with vessels to exert direct and dynamic control of local blood flow [20], [21]. Is cochlear blood flow controlled by an analogous mechanism?

This study is the first one to show that fibrocytes in the super-strial region are physically linked to vessels in the spiral ligament through extending processes. Elevation of Ca^2+^ in fibrocytes, induced by photolytic release of caged Ca^2+^, results in propagation of a Ca^2+^ signal in neighboring vascular cells. Ca^2+^-dependent release of a vasoactive compound, COX-1, causes capillary dilation, and both inhibition of COX1 and blockage of gap junctions attenuate acoustic-evoked vasoactivity. Our findings demonstrate the key role of fibrocyte–to-vascular cell signaled regulation of cochlear blood flow, particularly for meeting metabolic demand during increased sound activity. The experimental evidence supports a paradigmatic shift in which local regulation of cochlear blood flow has a larger role.

## Materials and Methods

### Ethics Statement

All procedures were reviewed and approved by the Institutional Animal Care and Use Committee at Oregon Health & Science University (IACUC approval number: B11265).

### Animals

Albino guinea pigs (CRL: Duncan-Hartley, both sexes, age 4–5 weeks, weight 300–450 g) were used in this study.

### Isolation of Whole Mounted Cochlear Lateral Wall Tissue

Guinea pigs were anesthetized with a 1 ml/kg intramuscular injection of ketamine 500 mg, xylazine 20 mg, and acepromazine 10 mg in 8.5 mL H_2_O, and then sacrificed by exsanguination. Both bullae were rapidly removed and transferred to a Petri dish filled with a physiological solution (in mM) of NaCl 125, KCl 3.5, glucose 5, HEPES 10, CaCl_2_ 1.3, MgCl_2_ 1.5, and NaH_2_PO_4_ 0.51 bubbled with 95% O_2_ and 5% CO_2_. The osmolarity of the solution was adjusted to 310 mOsm with NaCl, and the pH adjusted to 7.4 with NaOH. The spiral ligament was isolated, and the lateral wall of the cochlear second turn extracted. All experiments were performed at 37°C using a temperature-control chamber (Warner Instruments, Hamden, CT). Tissues were maintained in the physiological solution until needed.

### *In Vivo* Preparation

Guinea pigs were anesthetized with an injection of ketamine (40 mg/kg) and xylazine (10 mg/kg), and wrapped in a heating pad, with rectal temperature maintained at approximately 37°C. The head was fastened in a heated manipulator to prevent conductive cooling. The right jugular vein was cannulated for injection of fluorescent dye, and the contralateral carotid artery cannulated in a retrograde manner for continuous blood-pressure measurement. The left bulla was opened through a lateral and ventral approach, leaving the tympanic membrane and ossicles intact. To observe blood circulation in vessels of the spiral ligament, a rectangular fenestra (0.2×0.3 mm “vessel-window”) was made over the third turn by gently scoring and elevating the cochlear bony wall with a small knife blade [22], [23]. The vessel–window was cover-slipped to preserve normal physiological conditions and provide the best optical view for recording vessel images (see Fig. S2). By adjusting the optical focus, the fibrocytes and vessels of the spiral ligament were visualized. The vessels located in the window were continuously monitored with video-microscopy using a long working-distance objective lens (20×, NA 0.4). The images were recorded with a CCD camera at a rate of 30 frames/sec and digitally saved to a computer disk.

### Photolysis of Caged Calcium and Imaging of Calcium Signals *in Vitro* and *in Vivo*

The cochlear capillaries were pre-labeled with the fluorescent dye 1, 1-Dioctadecyl-3,3,3,3-tetramethylindocarbocyanine perchlorate, Dil [24] dissolved in DMSO (6 mg/ml). Immediately prior to IV infusion, the stock solution was diluted with phosphate buffered saline (PBS) to a final concentration of 3 mg/ml. One ml of the dye solution was slowly administrated intravenously to the guinea pig over a 5 min interval. For *in vitro* uncaging experiments, isolated segments of the cochlear lateral wall were incubated in *o*-nitrophenyl EGTA AM (a caged-Ca^2+^ probe, 10 µM, Invitrogen), pluronic acid (2.6 mg/ml), and fluo-4AM (10 µM, Invitrogen) for 30 min, and the tissues viewed with an FV1000 Olympus laser-scanning confocal microscope and 40× objective (NA 1.3). Fluo-4, used as a sensor for intracellular Ca^2+^, was excited at 488 nm and its fluorescence acquired through a 510 nm emission filter. Ca^2+^ in fibrocytes was photo-released with 600 nanosecond flashes of 405 nm laser light focused to a 5 µm spot. For *in vivo* uncaging experiments, the vessel-window was loaded with the same caged Ca^2+^ compound for 60 min. Intracellular Ca^2+^ was imaged on an Olympus BXFM fluorescence microscope equipped with a long-working-distant objective (20×, NA 0.4). Excitation at 375 nm, for photolysis of the Ca^2+^ cage, NP-EGTA, was obtained from a diode laser light source focused to a 10 µm spot. Images were captured by a Hamamatsu CCD camera, with the intracellular Ca^2+^ signals selected and analyzed on ImageJ software (NIH). The strength of the Ca^2+^ signal was assessed as a relative increase of fluorescence from baseline intensity (ΔF/F).

### Capillary Diameter and Blood Velocity Measurements

The internal (luminal) diameter of the capillaries was determined from acquired images as the distance between two fixed points across the capillary and directly adjacent to an identified fibrocyte end-foot using ImageJ [25]. Capillary diameter was measured at locations of maximum response. Constriction or dilation was presented either as a change in diameter or percentage of the baseline diameter. Blood velocity was determined from captured video frames and analyzed off-line. Blood flow velocity was calculated by a cross-correlation method using custom-made image analysis software.

### Sound Stimulation

A 500 Hz pure tone (a frequency optimal for the third turn vessel window) was applied in the external ear canal. Sound was administered at an intensity of 85 dB SPL. CBF was recorded for 3 min prior to sound stimulation, the last 3 minutes of the 10-min duration of sound stimulation, and for 3 additional minutes with the sound stimulation turned off. In the control group, the vessel-window was superfused with a perilymphatic solution for 10 min prior to sound stimulation and continued for the duration of the stimulus. In the inhibitory group, the vessel-window was superfused with a perilymphatic solution containing either the COX-1 inhibitor SC 560 for 10 min or the gap junction blocker CBX for 30 min by superfusion prior to sound stimulus and continued throughout the stimulus. A flow chart of the experimental sequence is shown in Fig. S3.

### Transmission Electron Microscopy

Cochlear lateral wall tissues were dissected and fixed overnight with phosphate buffered 3% glutaraldehyde-1.5% paraformaldehyde and postfixed in 1% osmium. Tissues were dehydrated and embedded in Araldite plastic, sectioned, stained with lead citrate and uranyl acetate, and viewed in a Philips EM 100 transmission electron microscope.

### Immunohistochemistry

The primary antibodies used in the experiments included anti-desmin (rabbit monoclonal to desmin, cat# ab32362, Abcam, Cambridge, MA), anti-collagen type IV (cat# ab6586, Abcam, Cambridge, MA), anti-COX1 (cat# Sc-1752, Santa Cruz Biotechnology, Inc., Santa Cruz, CA), anti-S100 (cat# ab8330, Abcam, Cambridge, MA), and anti-Na^+^/K^+^ ATPase β1 (cat# 06-170, Upstate, Lake Placid, NY).

Secondary antibodies (Invitrogen, Carlsbad, CA) included Alexa fluor 568 conjugate goat-anti-rabbit (cat# A11011), Alexa fluor 488 conjugate goat anti-rabbit (1∶100, cat# A11008, Invitrogen), Alexa fluor 488 conjugate goat anti-mouse IgG (H+L) (1∶100, cat# A11001), and Alexa fluor 568 conjugate rabbit anti-goat (cat# A11079).

Immunohistochemistry was performed as described previously [26]. Briefly, tissue sections were permeabilized in 0.5% Triton X-100 (Sigma, St. Louis, MO) for 1 h and immuno-blocked in a solution of 10% goat serum and 1% bovine serum albumin (BSA) in 0.02 M PBS for 1 h. The specimens were incubated overnight at 4°C with the primary antibody diluted in PBS-BSA. After several washes in PBS, the sections were incubated in a secondary antibody for 1 h at room temperature. Finally, after washes in PBS, the tissues were mounted with Slow Fade Light Antifade reagent (Invitrogen) and visualized with an Olympus Fluoview FV1000 confocal laser microscope system on an Olympus IX81 inverted frame. The controls were prepared by replacing primary antibodies with 0.2% Triton X-100 in PBS.

### Triple Labeling

To visualize the suprastrial structure of the cochlear-lateral wall, we triple labeled lateral-wall tissues with an antibody for desmin (or NG2) to identify pericytes (Abcam), isolectin GS-IB4 Alexa Fluor 647 to identify vessels (Invitrogen), and phalloidin-conjugated FITC to label the overall structure (Invitrogen). The procedure for immunohistochemically labeled desmin was the same as described above, except that 1∶400 isolectin GS-IB4 was added to the medium, along with the primary antibody for desmin.

### Double labeling

To visualize the spatial relationship between fibrocytes and capillaries, the whole mounted cochlear lateral-wall tissue was double labeled with an antibody for either S-100 or Na^+^/K^+^ ATPase β1 to identify the fibrocytes, and isolectin GS-IB4 Alexa Fluor 647 to identify the vessels (Invitrogen).

### Reverse Transcription Polymerase Chain Reaction

Total RNA from the cochlear lateral wall was separately extracted for each experimental group with a RNeasy kit (Qiagen, Valencia, CA) according to the manufacturer's suggestions. Each cohort of two mice was analyzed for COX mRNA. One µg of total RNA was reverse-transcribed using a RETROscript kit (Ambion, Austin, TX). Conserved regions spanning introns were selected for the primers of *Cox* and glyceraldehyde-3-phosphate dehydrogenase (*Gapdh*). The primers used were: *Cox1* (mouse Chr 2 NM_008969), forward; CATCCATCCACTCCCAGA, reverse; GAGGGCTGGGGATAAGGTTGG; 409-bp product; *Cox2* (mouse Chr 1 NM_011198), forward; GGGTTGCTGGGGGAAATGTG, reverse; GGTGGCTGTTTTGGTAGGTG; 479-bp; *Cox3* (mouse Chr 2 NM_008969), forward; CAGAGTCATGAGTCGTGAG, reverse; AGAGGGCAGAATGCGAGTAT; 584-bp; *Gapdh* (mouse Chr 6 NM_008084), forward; AACTTTGGCATTGTGGAAGG, reverse; ACACATTGGGGGTAGGAACA; 272-bp product. The RT-PCR was cycled at 95°C for 2 min, up to 40 cycles at 95°C for 30 sec, 60°C for 45 sec, 72°C for 30 sec, and a final 5-min extension at 72°C. The products of the reverse transcription polymerase chain reaction were visualized by agarose gel electrophoresis.

### DAF-2DA staining for NO

NO production was detected with the fluorescent indicator, diaminofluorescein – 2 diacetate (DAF-2DA), as previously described [27]. The auditory bulla was dissected and rapidly opened in a petri dish of physiological solution. Small pieces of tissue from the basal middle turn of the cochlear-lateral wall were removed, incubated in a physiological solution at 37°C, pH 7.4, containing 10 µmol/L DAF-2DA (cat^#^ 251505, Calbiochem, USA) for 30 min, and subsequently washed in fresh physiological solution for 10 min and imaged by confocal microscopy.

### COX Pathway Inhibition and Gap Junction Blockage

To determine whether the COX-1 pathway is involved in type V fibrocyte-capillary coupled signaling, tissues were pretreated with a specific COX-1 inhibitor, SC-560 [28], [29], [30]. To determine whether gap junctions are involved in the sound-induced Ca^2+^ signaling of fibrocytes, tissues were pretreated with a specific gap junction blocker, carbenoxolone (*CBX*; at 100 µM) [31], [32], [33]. The COX-1 inhibitor was added to the perfusion bath 10 min before photolysis and maintained in solution during photolysis. The gap junction blocker, CBX, was added to the perfusion bath, as well as directly applied to the round window, 30 min prior to sound stimulation (see Fig. S3).

### Measurement of COX-1 Enzymatic Activity

Anaesthetized animals were perfused with PBS to remove any red blood cells and sacrificed. The cochleae were removed from the auditory bullae immediately after sacrifice. Super-strial regions of the lateral wall were carefully separated from the cochleae, and the tissues incubated in a perilymphatic solution containing different concentrations of SC 560 (10^−7^ M, 10^−6^ M, 10^−5^ M, 10^−4^ M, 10^−3^ M, 10^−2^ M) for 30 min. Tissues were homogenized in 300 µL 0.1 M Tris-HCl buffer (pH 7.8) and centrifuged at 10,000 g for 15 minutes at 4°C. COX-1 activity of the supernatant was measured using a COX activity assay kit (Cat#760151, Cayman Chemical, Ann Arbor, MI) in the presence of a COX-2 inhibitor according to manufacturer's instructions.

### Statistics

Data are presented as means ± s.d. Statistical significance was determined by using the Student's *t* test (for two group comparisons) or ANOVA (for three or more groups). A 95% confidence level was considered statistically significant.

## Results

### Fibrocytes in the Super-strial Region Morphologically Couple with Capillaries

**Figure 1:**
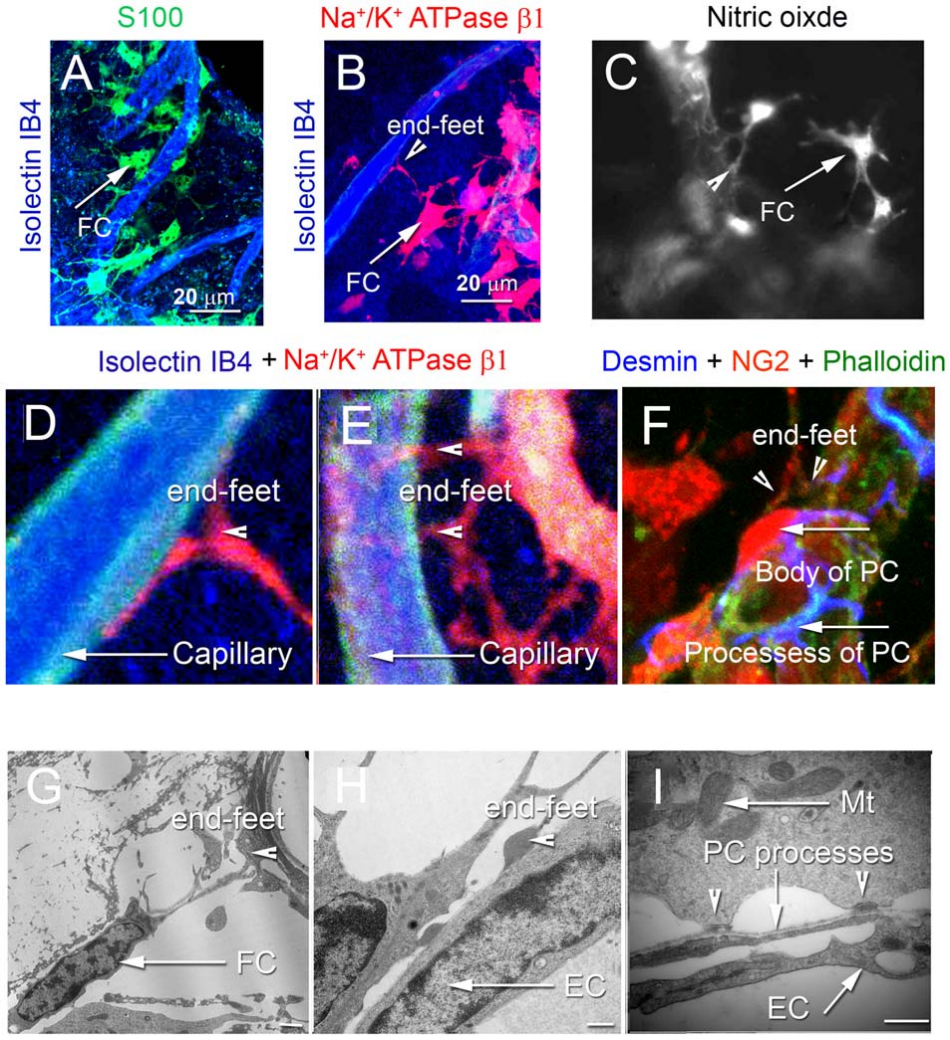
Fibro-vascular coupled morphology under confocal and TEM microscopy. (*A*) Type V fibrocytes positive for S100 (green) abut capillary walls labeled by isolectin IB4 (blue). (*B*) Type V fibrocytes are positive for Na^+^/K^+^ ATPase β1 (red). (*C*) Type V fibrocytes also contain high levels of NO, as detected with the intracellular NO indicator, DAF-2DA (gray). (*D*) Magnification of panel B shows foot processes in contact with a capillary. (*E*) A multiple-foot process of a fibrocyte abuts a capillary wall. (*F*) A high magnification image shows a fibrocyte end-foot structure at the soma of a pericyte. The somas of the pericytes were labeled by an antibody for NG2, (red), and processes were labeled with an antibody for the structural protein, desmin (blue).) Capillary walls are labeled by phalloidin (green). (*G*) and (*H*) Fibrocytes contact capillaries with enlarged endings. (*I*) The endings display electron-dense membrane regions rich in mitochondria. Abbreviations: FC, fibrocyte; EC, endothelial cells; PC, pericyte; Mt, mitochondria. Calibration bars in *H* and *I* are 500 nm.

Using confocal and transmission electron microscopy (TEM), we found some fibrocytes in the supra-strial region have interdigitating processes that abut the capillaries (Fig. 1). The coupled fibrocytes expressed S-100 protein (Fig. 1*A*), a calcium binding protein found in a wide range of mesenchymal cells, including fibrocytes, astrocytes [34], [35] and Na^+^/K^+^-ATPaseβ1 (Fig. 1*B*), a Na^+^/K^+^ pump that is also found in fibrocytes in other regions of the spiral ligament (Suko et al., 2000). In addition, the coupled fibrocytes exhibited significant nitric oxide (NO) production (Fig. 1*C*). At high magnification, fibrocytes are seen connected to microvessel walls through end-foot structures (Fig. 1*D*). Some fibrocytes connected to capillaries through one or more processes (Fig. 1*E*), while others directly connected to the body of pericytes through their processes (Fig. 1*F*). Under TEM, fibrocytes connected to capillaries were frequently found with enlarged endings (Fig. 1*G* and 1*H*). They also appeared to directly connect with capillaries through electron-dense membrane regions (Fig. 1*I*).

### *Ca^2+^* Signaling between Fibrocytes and Capillaries

**Figure 2:**
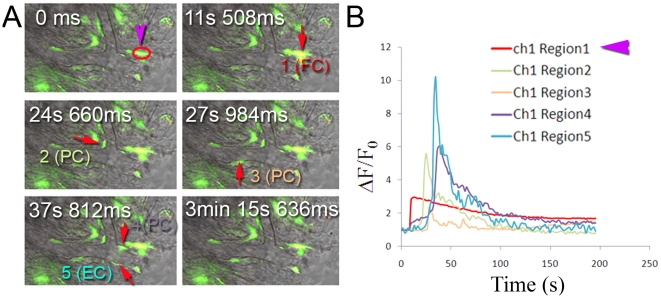
Photolysis of caged Ca^2+^ in fibrocytes initiates a propagating Ca^2^ wave in capillaries. (*A*) Fibrocytes communicate with nearby vascular cells. The fibrocyte was stimulated by photolysis at 1 (the purple arrow indicates the site of the uncaging flash). Note the photolysis-evoked Ca^2+^ wave (1 FC) propagates sequentially to vascular cells [2 (PC), 3 (PC), 4 (PC), and 5 (EC)]. (*B*) Ca^2+^ probe fluorescence from stimulation of fibrocytes propagates with delay to vascular cells.

Photolytic release of Ca^2+^ in stimulated fibrocytes *in vitro* evokes a calcium signal which propagates to neighboring vascular cells, including pericytes (PC) and endothelial cells (EC) positioned along capillary walls. The high resolution confocal images in Fig. 2*A*, imaged with DIC and fluorescence time-lapse, show Ca^2+^ communication between stimulated fibrocytes and vascular cells. Changes in fluorescence of the Ca^2+^ probe in the stimulated fibrocyte and, with delay, in the vascular cells are seen in the time course image of Fig. 2*B* [movie S1].

### Fibrocytes Regulate Capillary Diameter

An *in vivo* preparation of a vessel-window in the lateral wall was used to test whether fibrocyte activation affects capillary diameter. The vessel-window was made on the third turn of the cochlear lateral wall in a live animal (see Fig. S2 for more details). Systemic injection of the fluorescent dye Dil enabled visualization of the capillaries. The spiral ligament in the vessel-window was superfused with artificial perilymph and loaded with the Ca^2+^ indicator probe fluo-4. An intravital microscope with a long working distance objective enabled clear visualization of fluorescence in the fibrocytes. Fibrocytes with high intracellular calcium signals from the supra-strial region of the spiral ligament were selected for experimentation, as the connected fibrocytes display higher intracellular fluo-4 fluorescence than other cells in the cochlear lateral wall. The reason for this is unknown. Double fluorescence immunohistochemical staining showed the high fluo-4-AM fluorescent cells in the supra-strial region positive for S100, a marker protein for fibrocytes, but negative for desmin, a pericyte marker protein (see Fig. S4 and Fig. S5).

**Figure 3:**
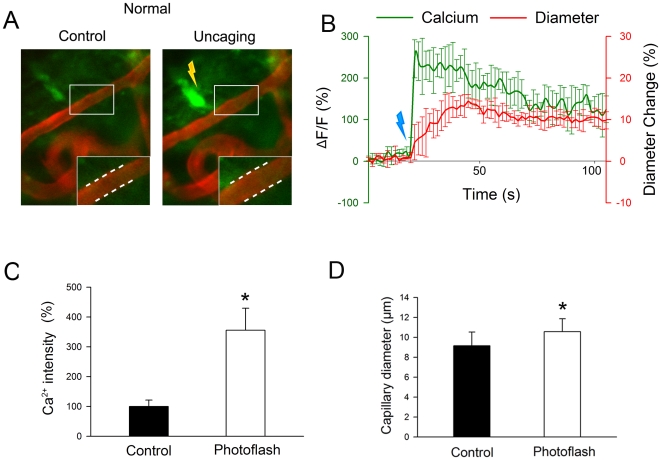
Photolysis of caged Ca^2+^ in fibrocytes evokes vasodilation *in vivo*. ((*A*) Photolysis-evoked vasodilation (*left*, before photolysis; *right*, after photolysis; white dotted lines in A, B indicate sites of dilation). (*B*) Photolysis-evoked, time-dependent change in intracellular Ca^2+^ in stimulated fibrocyte (green line) correlates with the change in capillary diameter (red line). (*C*) Mean fluorescent signal of the Ca^2+^ indicator is significantly increased. (*D*) Mean capillary diameter is significantly increased (n = 8, P<0.01).

Ca^2+^ was elevated by photolytically uncaging EGTA-AM with UV excitation spatially targeted to 10 µm spots in the supra-strial region. High Ca^2+^ signaling in stimulated fibrocytes is associated with dilation of vessels (Fig. 3 *B* and Fig. 3*C*). We found that ∼53% of stimulated fibrocytes resulted in dilated vessels. When dilation occurred, capillary diameter increased ∼15% (Fig. 3*C*, before: 9.15±1.25 µm; after: 10.55±1.36 µm, n = 8, P<0.01).

### COX-1 Metabolites Required for Vasodilation

**Figure 4:**
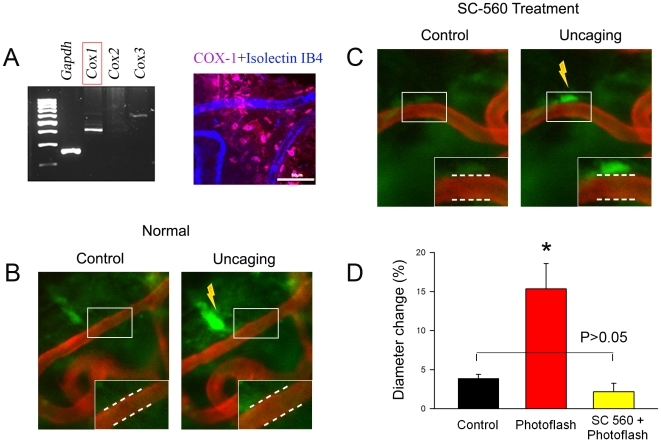
Photolysis of caged Ca^2+^ in fibrocytes evokes vasodiation in *vivo* through COX-1 signaling. (*A*) mRNA for *Cox-1* and *Cox-3* is expressed in the cochlear lateral wall (*left*). COX-1 protein is selectively expressed in type V fibrocytes, but not in vascular cells (*right*). (B) Photolysis evokes vasodilation (l*eft*, before photolysis; *right*, after photolysis; white dotted lines in l*eft*, *right* indicates sites of dilation). (*C*) Lack of photolysis-evoked vasodilation is shown (l*eft*, before photolysis; *right*, after photolysis; white dotted lines in l*eft*, *right* indicates sites of changes of capillary diameter). (*D*) Mean capillary diameter is significantly increased before and after photolysis (n = 8, P<0.01). In contrast, mean capillary diameter is unchanged in tissues treated with a COX-1 inhibitor (n = 10, P>0.05).

We hypothesize that fibrocytes and vascular cells are coupled at the fibro-vascular interface by local metabolic signals *in vivo*. The COX signaling pathway for modulating capillary diameter was specifically tested, as the pathway is known to be important in regulating microvessel diameter in brain and retina [36], [37]. Using RT-PCR, we found mRNA for *Cox-1* and *Cox-3* expressed in the cochlear lateral wall, with particularly high expression of COX-1 (Fig. 4*A*, *left*). Immunostaining revealed the COX-1 selectively expressed in type V fibrocytes, but not in vascular cells (Fig. 4*A*, *right*). The functional relevance of the signaling was tested by inhibiting the COX-1 pathway. Photolysis of fibrocytes in a “vessel-window” superfused with perilymphatic solution was associated with the dilation of vessels (Fig.4*B*). In contrast, superfusion of the “vessel-window” with a perilymphatic solution containing a specific inhibitor of COX-1, SC-560, at 500 µM for 10 min (applied concentrations were based on an *in vitro* dose-response of COX-1 by SC-560, see Fig. S6) before photolysis blocked photolysis-evoked vasodilation (Fig. 4*C*). Change in mean capillary diameter was reduced (Fig. 4*D*, 2.2%±1.1%, n = 10, P = 0.11>0.05). The results link COX-1 to regulation of regional blood flow.

### Fibro-Vascular Coupled Mediation of Sound-induced CBF

Fibro-vascular units functionally “bridge” between increased sound activity and CBF *in vivo*. In our model, fibro-vascular coupled units integrate the mechanical energy of sound, initiate Ca^2+^ and COX-1 signaling, and affect CBF.

**Figure 5:**
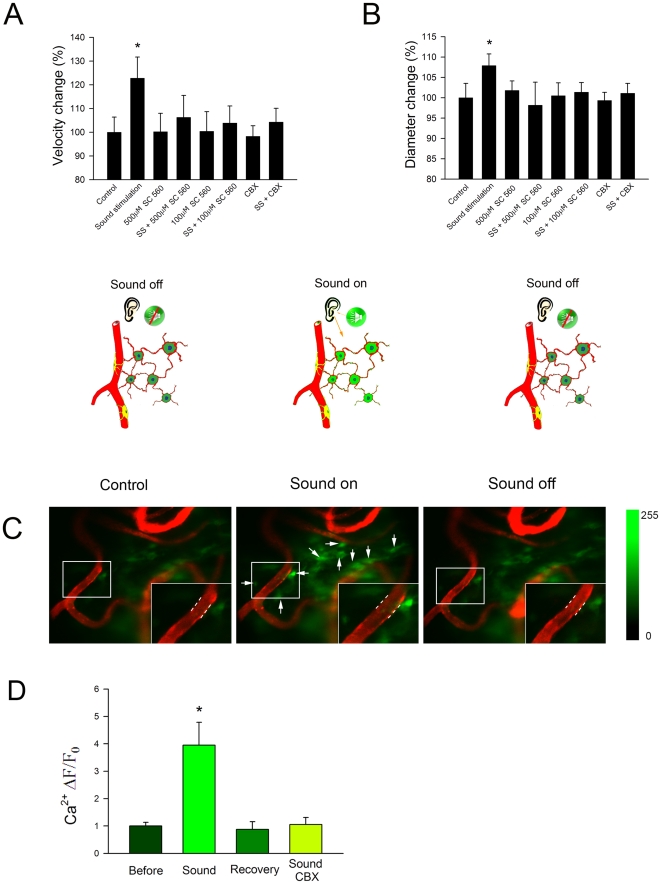
Sound-induced changes of intracellular Ca^2+^ in fibrocytes, blood-flow velocity, and capillary diameter. (*A*) and (*B*) Changes in cochlear blood flow velocity and capillary diameter under a variety of conditions: control, sound stimulated, COX-1 inhibited, and sound stimulated with COX-1 and CBX inhibition. Sound stimulation alone caused significant increases in capillary diameter and blood-flow velocity (n = 15, P<0.05). However, prior perfusion of the vessel-window with the COX-1-specific inhibitor, SC 560, or with the gap junction blocker, CBX , essentially blocked the sound-induced dilation. The cartoon shows the sound-stimulation protocol. (*C*) Intracellular Ca^2+^ signals are shown under control (left, no sound) and sound-stimulated conditions (middle, sound on). Fluorescence of the intracellular Ca^2+^ probe in some fibrocytes (arrows) returns to normal about 2 min after sound stimulation (right, sound off). (*D*) Mean Ca^2+^ signal was significantly higher in the sound stimulated fibrocytes.

Sound stimulation applied to the inner ear causes Ca^2+^ signaling in fibrocytes. In these experiments blood flow was recorded of the vessel-window preparation with a CCD camera for 3 min prior to sound stimulation to establish a baseline, 10 min with sound stimulation (500 Hz pure tone at 85 dB SPL applied to the external ear), and for an additional 3 min following sound stimulation. Tissues in the vessel-window were superfused with fluo 4, a Ca^2+^ indicator, and intravenously pre-labeled with Dil. A flow chart of the experimental sequence is provided in Fig. S3. Consistent with previous reports [1], sound stimulation increased both blood flow velocity (Δ velocity = 22.7%, n = 15, P<0.05, Fig. 5*A*) and capillary diameter (Δ diameter = 7.9%, n = 15, P<0.05, Fig. 5*B*). In addition, sound caused a significant increase in Ca^2+^ signaling in fibrocytes (Fig. 5*C*, *middle*). A plot of the normalized sound stimulated Ca^2+^ indicator signal is shown in Fig. 5*D*.

Fibrocytes participate in ion transport and facilitate generation of the EP by recycling K^+^ from hair cells to intermediate cells in the stria vascularis through gap junctions [13], [18], [19]. It follows that increased hair-cell activity must be matched by increased fibrocyte metabolism. Involvement of fibro-vascular coupled signaling was tested by blocking the gap junctions between epithelial cells. In support of our conjecture, pre-treatment of the cochlea with a specific blocker (CBX applied at 100 µM to the round window for 30 min, combined with a superfusion of perilymphatic solution containing CBX at 100 µM before and during sound stimulation) blocked sound-evoked Ca^2+^ signaling (Fig. 5*D*) and eliminated the vasodilative response to sound (Fig. 5*A* and 5*B*).

COX-1 was shown to be the downstream signal responsible for sound induced capillary dilation. Superfusion of the vessel-window with a perilymphatic solution containing a specific inhibitor of COX-1, SC-560, at 100 µM and 500 µM for 10 min before sound stimulation abolished sound-induced vessel dilation and eliminates changes in blood flow velocity (Fig. 5*A* and 5*B*). The result is consistent with results obtained from photolysis of caged Ca^2+^ (Fig. 4*D*).

The experimental findings support a model in which acoustic stimulation elevates fibrocyte Ca^2+^, initiates fibrocyte-to-fibrocyte signaling, induces release of vasoactive compounds, and causes vessel dilation.

## Discussion

This study provides the first evidence that cochlear fibrocytes, “activated” by sound, mediate control of capillary diameter and blood flow in the inner ear. The evidence suggests fibrocyte to vascular cell signaling is a key mechanism modulating CBF in response to sound.

Fibrocytes have long been regarded as simple supporting cells; however, recent evidence suggests fibrocytes have other functional roles under both physiological and pathological conditions [15], [38], [39], [40], [41], [42], [43], [44], [45], [46], [47]. For example, fibrocytes participate in ion transport, facilitating generation of the endocochlear potential by recycling K^+^ from hair cell transduction.

Fibrocytes in the cochlear lateral wall are classified as types I to V based on morphological appearance, staining pattern, and general location [15], [16] (also see Fig. S1). In general, type I fibrocytes lie behind the stria vascularis and follow the curvature of the lateral wall, while type II fibrocytes lie toward the scala tympani side of the stria vascularis, and type III fibrocytes circumferentially line the otic capsule. Type IV fibrocytes are spindle-shaped and lateral to the basilar membrane, while type V fibrocytes lie above the stria vascularis (supra-strial area) where arterioles branch into precapillaries and into the two true capillary networks, the capillaries of the spiral ligament and stria vascularis [10]. In the supra-strial region pre-capillaries contain a high population of pericytes. Pericytes on pre-capillaries are spaced approximately 2–25 µm apart, compared to up to 100 µm on true capillaries [8]. Pericyte contraction and dilation can significantly affect capillary diameter [9], [48]. Pericyte contraction would significantly affect the flow resistance of the vascular network, and profoundly impact overall blood flow.

In this study, we classified these capillary-coupled cells as fibrocytes, based on the fibrocyte identification described by Spicer and Schulte [15] as being immunohistochemically positive for S-100 and Na^+^/K^+^-ATPaseβ1 proteins, and exclusive of macrophages and pericytes (Fig. S1). However, this identification does not conclusively exclude other cell types, as the complexity of the lateral wall and lack of specific markers differentiating between fibrocytes and other cell types of the same lineage make definitive classification difficult.

In addition to expressing S-100 and Na^+^/K^+^-ATPaseβ1 proteins, we found the connected fibrocytes display higher fluo-4 indicator fluorescence than other cells in the cochlear lateral wall under *in vivo* conditions (see Fig. S4). A majority of the high fluo-4 fluorescence cells were S-100 positive. In our *in vivo* photolysis study, high fluo-4 fluorescence was used to identify coupled fibrocytes.

**Figure 6:**
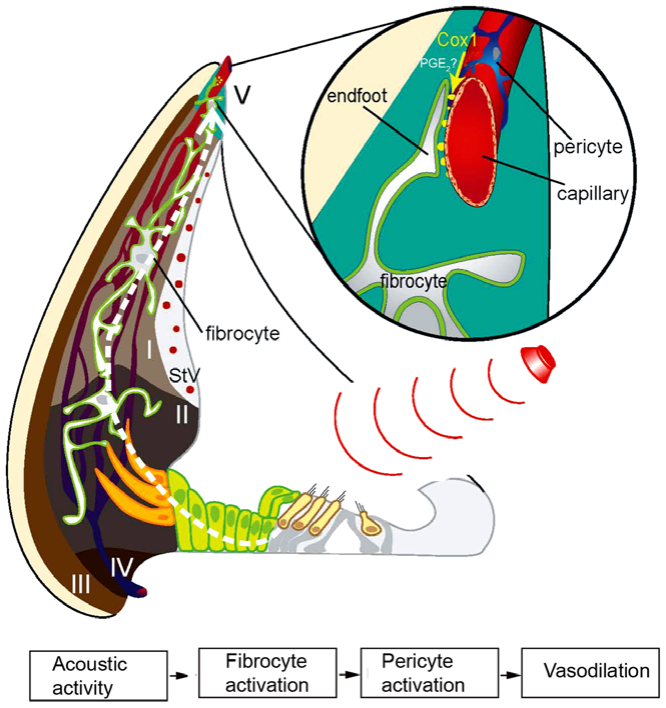
A working model of fibro-vascular coupled signaling in the inner ear. The schematic diagram illustrates selected aspects of fibrocyte signaling. Sound stimulation (red dotted line) activates hair cells and initiates Ca^2+^ signaling in fibrocytes. While the gating mechanisms for the Ca^2+^ signaling have not been determined, mechanical vibration or metabolic activity, such as K^+^ recycling, initiated by sound might underlie the gating. COX-1 is regulated by the elevation of Ca^2+^ in fibrocytes. The COX-1 converts arachidonic acid into metabolic intermediates, including PGE_2_, which diffuse into the perivascular space to elicit vasodilatation.

Changes in capillary diameter may directly result from pericyte action (relaxation or contraction) or indirectly result from action mediated by endothelial cells. In the case of indirect action, endothelial cells would release vasodilatory factors, such as endothelium-derived relaxing factor, to activate pericytes. In our study, we found approximately half of *in vivo* stimulated fibrocytes to dilate vessels. When dilation occurred, capillary diameter increased ∼15%. In contrast, only very occasional vasocontraction was seen in stimulated *in vitro* preparations. In addition, propagation of the Ca^2+^ signal between stimulated fibrocyte and vascular cells had a slightly longer latency period *in vitro* than *in vivo*. The discrepancy in vasomotor response could have been the result of experimental conditions, as vessels in the *in vitro* preparation lack the influence of intravascular pressure and flow. Also, *in vitro* vessel preparations usually do not develop spontaneous tone, and relaxation responses can only be studied after capillary preconstruction [49]. However, despite the lack of contraction, Ca^2+^ signaling between fibrocytes and capillaries *w*as still clearly observed *in vitro* (see Movie S1). Sound-initiated changes in Ca^2+^ signaling in fibrocytes underlie the functional interaction between fibrocytes and vascular cells. The gating mechanisms for the signaling are not clear, but both mechanical stretch and metabolite effects may be involved. The metabolic gating could also relate to the energy expenditure required for K^+^ recycling, as blockage of gap junctions eliminates normal sound-induced vasodilation. A working model is illustrated in Fig. 6

COX-1 activity is central to fibro-vascular coupled signaling in the cochlea, as pre-incubation with a selective COX-1 inhibitor, SC-560, blocks both photolytically released Ca^2+^ and sound induced vessel dilation in the cochlea *in vivo*. COX-1 activation may be analogous to COX -dependent vascular regulation in brain [50]. COX-1 is a rate-limiting enzyme which converts arachidonic acid into prostaglandins [30]. Elevation of Ca^2+^ increases COX activity and mobilizes arachidonic acid. COX then converts arachidonic acid to a prostaglandin such as PGE_2_ [51]. Blocking the COX pathway is shown to deregulate vasoactivity in several organ systems [52], [53]. However, more direct evidence on COX-1 metabolites is needed before firm conclusions can be drawn. Nevertheless, the COX-1 product involved in regional CBF regulation is a result consistent with the results of others.

No significant capillary diameter and blood flow velocity reduction was found after SC-560 administration in the control, no-sound condition (see Figure 5). Fibrocytes, including type V fibrocytes, participate in ion transport, which is essential for generating the endocochlear potential required for hearing. Increases in hair cell activity must be accompanied by increases in fibrocyte metabolism, as fibrocytes are involved in dynamic K^+^ recycling and in maintaining the endolymphatic potential needed for hair cell sensitivity. Overall, our data indicate that COX-1 activity is the link between metabolic need and local blood flow.

In summary, regulation of blood flow in response to acoustic activity is complex, since local processes controlling capillary flow interact with central control exerted through direct innervation of upstream vessels. There's also evidence multiple metabolic factors, including ATP, NO, and K^+^, mediate CBF, as fibrocytes produce NO and express both S-100, a Ca^2+^-binding protein, and Na^+^/K^+^-ATPase β1. Despite these complicating other factors, however, this study establishes for the first time a physiological link between fibrocytes and cochlear vessels which underlies the response to sound. We were able to elucidate the role of fibrocytes in controlling local blood flow experimentally by using targeted *in vivo* and *in vitro* stimulation. We showed that flow-metabolic coupled signaling is a key mechanism in modulating CBF to meet the metabolic demand that results from transduction of sound.

## Supporting Information

Figure S1 **Cochlear lateral wall structure from confocal fluorescence images of the suprastrial region.** (*A*) The drawing shows the location of type I–V fibrocytes in the cochlear lateral wall. (*B*) shows the organization of the suprastrial region. Tissue was labeled with phalloidin for F-actin (green). The region is rich in type V fibrocytes, pre-capillaries, and capillaries. (*C*) is an image of pre-capillaries and capillaries labeled with isolection IB4. (*D*) shows pericytes on the pre-capillaries and capillaries of the spiral ligament which have been labeled with an antibody for desmin, a marker for pericytes (red). (*E*) is a merged image from Panels A, B, & C which shows the supra stria vascularis region composed of pericyte-containing pre-capillaries, capillaries, and surrounding fibrocytes. (The white line indicates the location of Reissner's membrane, while the area below and to the right of the line is the suprastrial region.) Calibration bar with ticks is 50 µm.
(TIF)

Figure S2 **Illustration of the vessel-window perfusion system.** Fluids are delivered under the coverslip by a microtube connected to a manifold. This allows selection of the solution to be perfused without any delay for clearance of tubing. Perfusion is accomplished with a syringe pump. Fluid is wicked away from the cochlea with cotton.
(TIF)

Figure S3 **Flow chart of the experimental sequence.**
(TIF)

Figure S4 **Fluo-4 fluourescence in cells of the lateral wall.** Fibrocytes within a vessel-window of the super-strial region displayed a higher intracellular signal of the fluorescent Ca^2+^ indicator fluo-4 than other cells in the cochlear lateral wall. Capillaries were labeled with the fluorescent dye Dil.
(TIF)

Figure S5 **High fluo-4 fluorescence cells are fibrocytes.** Spiral ligament tissue in the vessel-window triple labeled with fluo-4 (green), S-100 antibody (red), and desmin antibody (blue) verifies a majority of high fluo-4-fluorescence cells (A, green) were positive for S-100, a fibrocyte marker protein (B), but negative for desmin, a pericyte marker protein (C). (D) A merged image from Panels A , B, and C. (E) A DIC image shows the capillaries located in the super-strial region. (F) A merged image of Panels A, B, C, D, and E.
(TIF)

Figure S6 (A) shows the dose-dependent inhibition of COX-1 by SC560 (data are expressed as mean ± SEM, n = 3 for each treatment). The IC50 for SC-560 in cochlear tissue is about 1 µM. Complete inhibition occurred at a concentration of ∼100 µM. (B & C) show the effect of different concentrations of SC-560 on blood flow velocity and capillary diameter.
(TIF)

Movie S1 **Photolysis-evoked Ca^2+^ signaling between fibrocytes and vascular cells.** The photolysis-evoked Ca^2+^ wave (1 FC) propagates sequentially to vascular cells [2 (PC), 3 (PC), 4 (PC), and 5 (EC). UV light stimulation is indicated by the purple arrow.
(AVI)
