# Supplementary figures and images for: Fibro-Vascular Coupling in the Control of Cochlear Blood Flow
Source: PLoS One. 2011 Jun 1;6(6):e20652. doi: 10.1371/journal.pone.0020652 (PMC3106013; doi:10.1371/journal.pone.0020652)

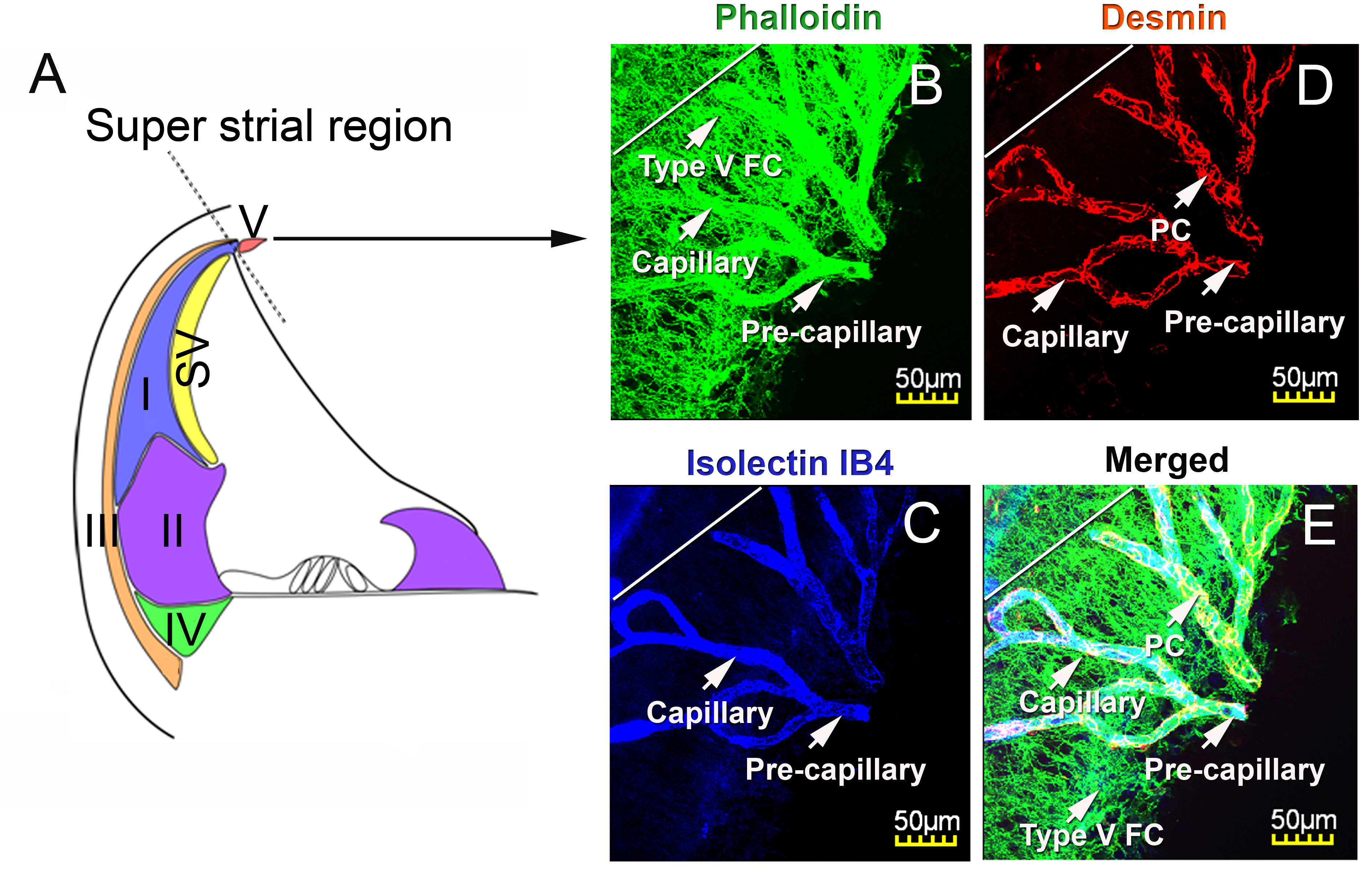

Supplement: Figure S1 — Cochlear lateral wall structure from confocal fluorescence images of the suprastrial region. (A) The drawing shows the location of type I–V fibrocytes in the cochlear lateral wall. (B) shows the organization of the suprastrial region. Tissue was labeled with phalloidin for F-actin (green). The region is rich in type V fibrocytes, pre-capillaries, and capillaries. (C) is an image of pre-capillaries and capillaries labeled with isolection IB4. (D) shows pericytes on the pre-capillaries and capillaries of the spiral ligament which have been labeled with an antibody for desmin, a marker for pericytes (red). (E) is a merged image from Panels A, B, & C which shows the supra stria vascularis region composed of pericyte-containing pre-capillaries, capillaries, and surrounding fibrocytes. (The white line indicates the location of Reissner's membrane, while the area below and to the right of the line is the suprastrial region.) Calibration bar with ticks is 50 µm. (TIF) [file pone.0020652.s001.tif]

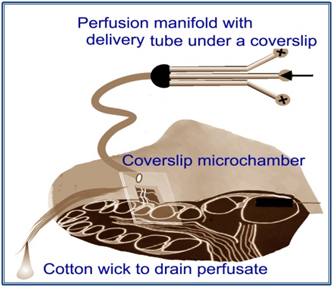

Supplement: Figure S2 — Illustration of the vessel-window perfusion system. Fluids are delivered under the coverslip by a microtube connected to a manifold. This allows selection of the solution to be perfused without any delay for clearance of tubing. Perfusion is accomplished with a syringe pump. Fluid is wicked away from the cochlea with cotton. (TIF) [file pone.0020652.s002.tif]

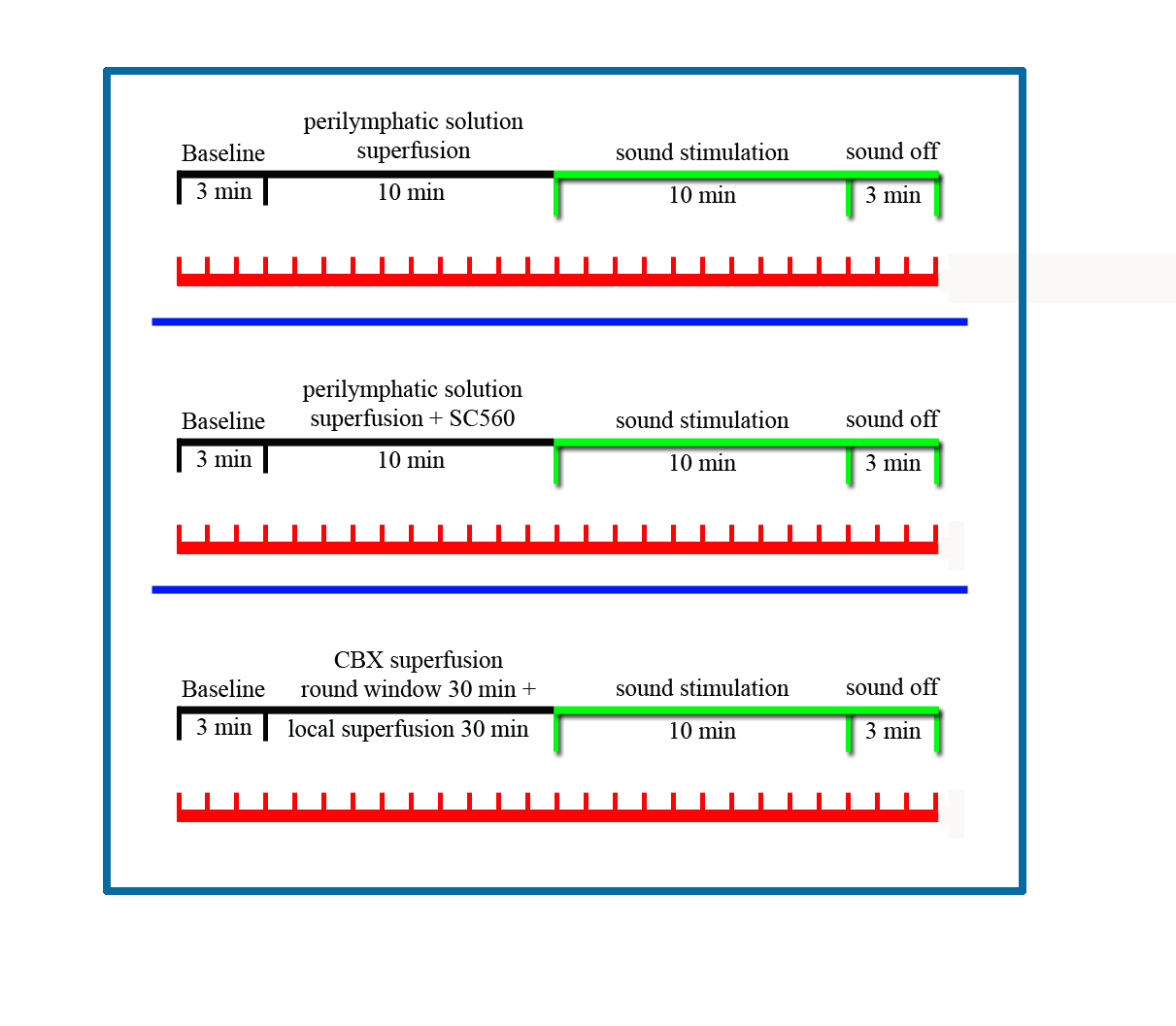

Supplement: Figure S3 — Flow chart of the experimental sequence. (TIF) [file pone.0020652.s003.tif]

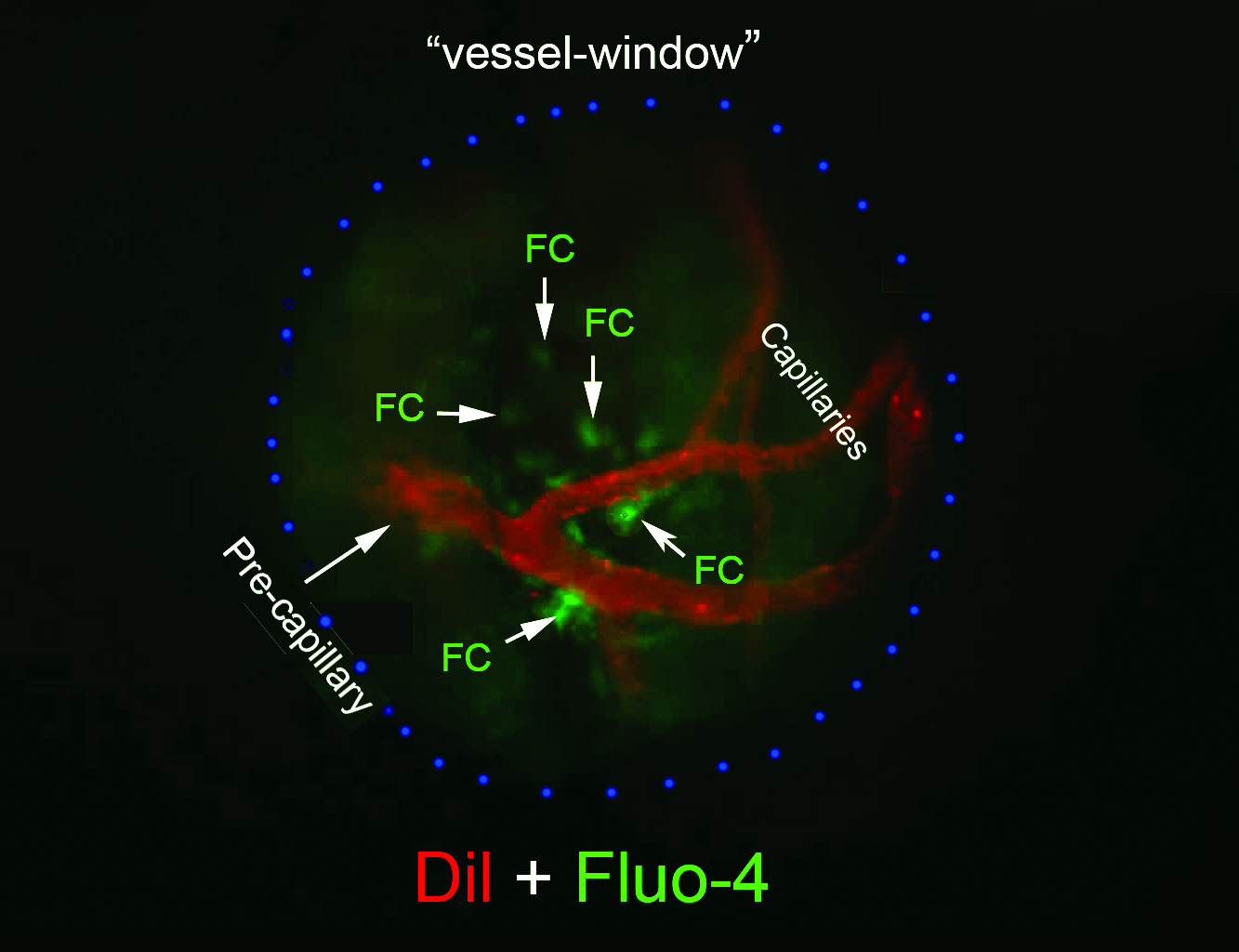

Supplement: Figure S4 — Fluo-4 fluourescence in cells of the lateral wall. Fibrocytes within a vessel-window of the super-strial region displayed a higher intracellular signal of the fluorescent Ca2+ indicator fluo-4 than other cells in the cochlear lateral wall. Capillaries were labeled with the fluorescent dye Dil. (TIF) [file pone.0020652.s004.tif]

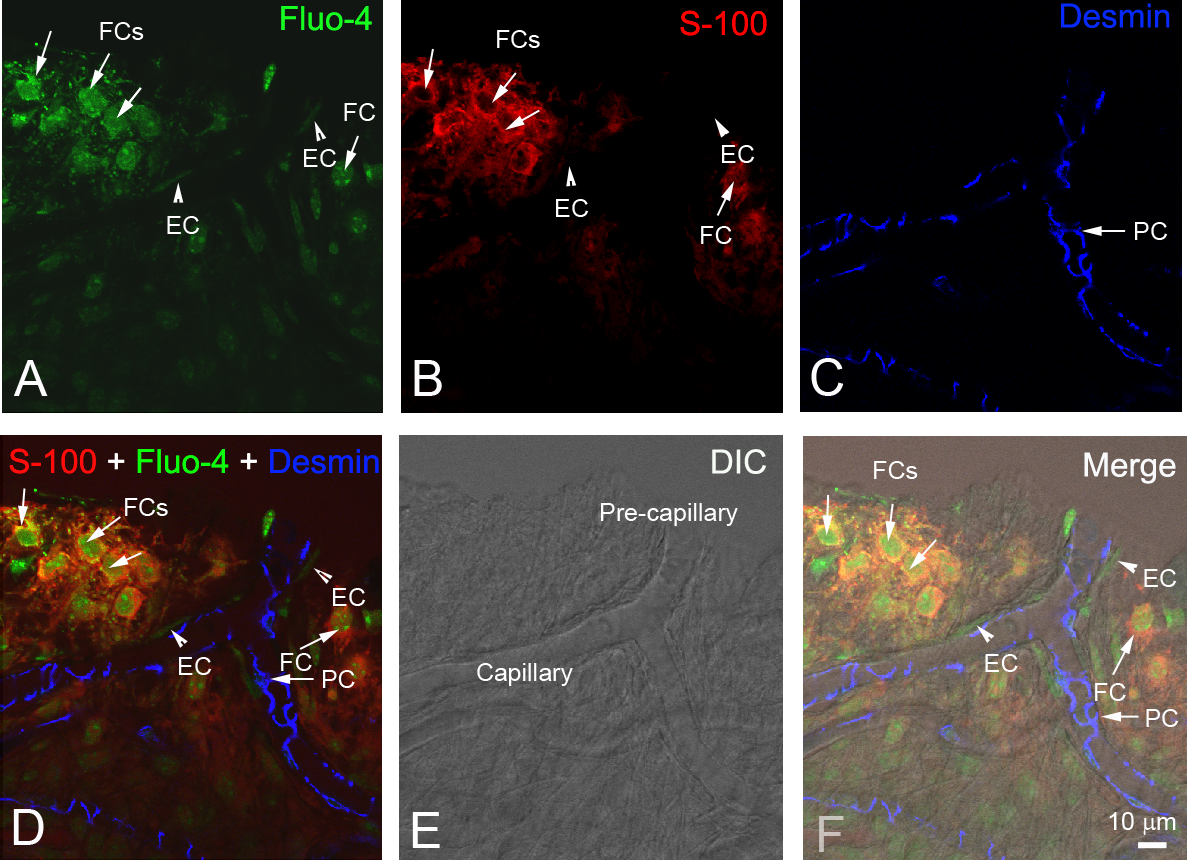

Supplement: Figure S5 — High fluo-4 fluorescence cells are fibrocytes. Spiral ligament tissue in the vessel-window triple labeled with fluo-4 (green), S-100 antibody (red), and desmin antibody (blue) verifies a majority of high fluo-4-fluorescence cells (A, green) were positive for S-100, a fibrocyte marker protein (B), but negative for desmin, a pericyte marker protein (C). (D) A merged image from Panels A , B, and C. (E) A DIC image shows the capillaries located in the super-strial region. (F) A merged image of Panels A, B, C, D, and E. (TIF) [file pone.0020652.s005.tif]

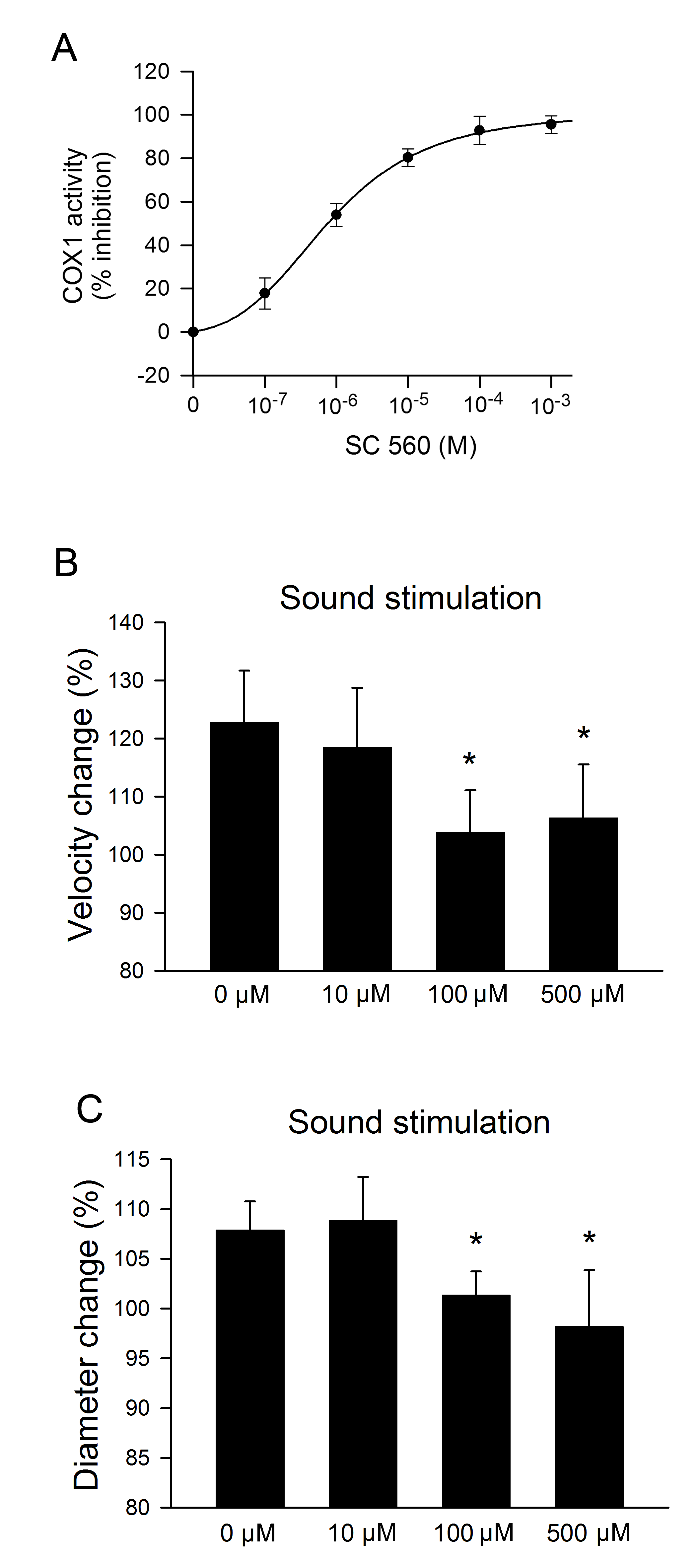

Supplement: Figure S6 — (A) shows the dose-dependent inhibition of COX-1 by SC560 (data are expressed as mean ± SEM, n = 3 for each treatment). The IC50 for SC-560 in cochlear tissue is about 1 µM. Complete inhibition occurred at a concentration of ∼100 µM. (B & C) show the effect of different concentrations of SC-560 on blood flow velocity and capillary diameter. (TIF) [file pone.0020652.s006.tif]
